# Exploring Taurodontism Using Cone Beam Computed Tomography: A Retrospective Study of Its Morphology and Association with C-Shaped Canal 

**DOI:** 10.30476/dentjods.2025.103867.2484

**Published:** 2025-12-01

**Authors:** Bahare Davvaz, Sasan Hosseini, Yasamin Ghahramani, Abdolaziz Haghnegahdar

**Affiliations:** 1 Resident, Dept. of Pediatric Dentistry, Faculty of Dentistry, Zahedan University of Medical Sciences, Zahedan, Iran.; 2 Student Research Committee, Dental School, Shiraz University of Medical Sciences, Shiraz, Iran.; 3 Dept. of Endodontics, Dental School, Shiraz University of Medical Sciences, Shiraz, Iran.; 4 Dept. of Oral and Maxillofacial Radiology, Dental School, Shiraz University of Medical Sciences, Shiraz, Iran.

**Keywords:** Taurodontism, C-shaped configuration, Dental malformations, Cone-beam computed tomography

## Abstract

**Background:**

Taurodontic teeth are known for complex root canal configurations, including C-shaped canals, further complicating dental procedures. Given the diagnostic and therapeutic challenges posed by taurodontism, understanding its prevalence and its relationship with canal morphology, especially through modern imaging techniques, is essential for improving clinical outcomes.

**Purpose:**

This study aimed to investigate taurodontism and explore its association with C-shaped canals using CBCT images from 2020 to 2024. This enhanced imaging technology will provide a more detailed view than panoramic imaging.

**Materials and Method:**

This retrospective analytical study examined 700 jaws (350 lower and 350 upper), chosen through simple random sampling from CBCT images originally taken for therapeutic purposes in individuals aged 14 and above. Teeth were evaluated based on Shifman and Chanannel's criteria and categorized as hypotaurodontism, mesotaurodontism, or hypertaurodontism according to morphology. Additionally, the study investigated the presence and types of C-shaped canals in the teeth.

**Results:**

In this study, the median age of the cases was 40 years, with 63.7% female and 36.3% male. The overall prevalence of taurodontism in teeth, regardless of jaw and tooth type, was 9%. Taurodontism was predominantly bilateral (84.7%) and mild (75%). It was significantly more common in maxillary teeth than mandibular teeth (*p*< 0.001), with the highest prevalence in the second and first maxillary molar teeth. Sex did not show a significant association with taurodontism prevalence (*p*= 0.208), type (*p*= 0.371), laterality (*p*= 0.627), as well as the involved tooth. Additionally, the prevalence of C-shaped canals was 15.4%, and it was significantly higher in individuals with taurodontism (*p*< 0.001).

**Conclusion:**

Taurodontism was found to be relatively common in the patients studied, with a high prevalence. Due to the challenging nature of treating taurodontic teeth, practitioners should be aware of the potential presence of C-shaped canals when encountering taurodontism in radiographic images.

## Introduction

Taurodontism is a dental defect characterized by smaller roots and larger tooth bodies, leading to enlarged pulp chambers and displaced roots. This condition, known for nearly a century, results in teeth with reduced roots and furcation areas near the root tips [ 1 ]. Sir Arthur Keith [ 2 ] first defined it in 1913, and Witkop's definition highlights enlarged pulp chambers, apically displaced furcations, and the absence of a typical constriction at the cementoenamel junction (CEJ) in taurodontic teeth [ 3 - 4 ].

Taurodontism is a common dental anomaly in humans with lower prevalence in Jewish and German communities and higher rates in Turkish, Iranian, and Chinese populations [ 5 - 9 ]. Epidemiological studies indicate that taurodontism is less prevalent in mandibular molars (2%) than in maxillary molars, with prevalence rates between 43% and 67%. This suggests that maxillary molars are more commonly affected by the condition [ 10 - 11 ]. Excluding third molars in some studies could result in underreporting of taurodontism, as these teeth may also exhibit the anomaly, with some studies indicating higher rates in third molars [ 12 ]. The prevalence of taurodontism varies significantly among different populations and tooth types, underscoring the need for further research to fully comprehend its distribution and implications. The pathophysiology of taurodontism involves theories linking it to odontoblastic deficiency and the failure of Hertwig's epithelial root sheath invagination during tooth development, resulting in enlarged pulp chambers [ 3 ]. Other hypotheses suggest delayed calcification of the pulp chamber and disruptions in developmental homeostasis as contributing factors [ 13 - 14 ].

The etiology of taurodontism includes the hypotheses that suggest it may be a primitive pattern, a mutation, or an autosomal dominant trait with familial links, and while it often occurs as an isolated anomaly, it can also be associated with developmental syndromes. Taurodontism presents significant clinical implications for various dental treatments, affecting endodontic, prosthetic, and orthodontic procedures [ 15 - 16 ]. In 1928, Shaw [ 17 ] introduced a classification system for taurodontism based on the relative displacement of the pulp chamber floor, categorizing it into three subtypes including (1) Hypotaurodontism(mild form with an enlarged pulp chamber), (2) Mesotaurodontism(moderate form with roots divided at the middle third), and (3) Hypertaurodontism(severe form with bifurcation or trifurcation near root apices). Another classification by Feichtinger and Rossiwall [ 18 ] in 1977 considered the distance from root bifurcation/trifurcation to the CEJ, which should exceed the occluso-cervical distance for a tooth to be classified as taurodontism.

Radiological examination is essential for identifying taurodontism, as it reveals unique features such as a rectangular tooth shape, enlarged pulp chamber, shorter roots, and atypical bifurcation locations, despite normal external morphology [ 19 ]. Initially, periapical radiographs was commonly used for detection, with manual measurements taken [ 5 ]. Panoramic radiographs later became more popular, offering a broader view of the dental arch. Researchers like Tulensalo *et al*. [ 20 ] were pioneers in utilizing panoramic radiographs for manual measurements. Subsequent studies, such as a Chinese study, utilized digital measurements from panoramic radiographs for more precise recordings [ 6 ]. The Taurodont Index (TI) became popular for its independence from the magnification inherent in panoramic radiographs [ 21 ].

The C-shaped canal configuration features a distinctive C-shaped cross-section and is typically found in mandibular second molars, though it can occur in other teeth. This anatomical variation poses challenges during root canal therapy due to the differing number and location of canals [ 22 ]. The etiology of the C-shaped canal anatomy is believed to be the failure of the fusion of Hertwig's epithelial root sheath, resulting in the formation of a lingual or buccal groove and a thin interradicular ribbon connecting the roots [ 23 - 24 ].

C-shaped canal configurations are more prevalent in certain ethnic populations, such as East Asian, South Asian, and Middle Eastern populations [ 22 , 25 - 26 ]. They are commonly found in mandibular second molars and can also occur in other teeth. The occurrence of C-shaped canals does not seem to be correlated with sex or age [ 22 , 25 - 26 ]. Recent studies have shown a high correlation between taurodontism and C-shaped canal configurations [ 27 - 28 ]. In cases where taurodontism and C-shaped canal configurations coexist, caution is needed during treatment due to the complexity of the canal system. Understanding these variations is essential for precise diagnosis and treatment planning. Further research is required to investigate the prevalence, morphology of taurodontism, and the relationship between taurodontism and C-shaped canals. This study aimed to investigate taurodontism and explore its association with C-shaped canals using CBCT images from 2020 to 2024. This enhanced imaging technology will provide a more detailed view than panoramic imaging. 

## Materials and Method

This study was carried out after obtaining ethical approval from the Ethical Committee of Dental School (Code: IR.SUMS.DENTAL.REC.1402.114). The study aimed for a sample size of 700 individuals determined through consultation with a statistical advisor using the Cochran formula. Samples were collected via simple random sampling from CBCT images in the Radiology Department archive of the Dental School, taken for therapeutic purposes in individuals over 14 years old. The CBCT images were prepared using NewTom VGi (QR-SRL, Venora, Italy) with a voxel resolution of 0.mm (10 mA, 110 kVp), a 1.8-second scan, and a field of view of at least 10×5. They were transferred to the NNTviewer software (NewTom, Venora, Italy). Exclusion criteria were defined as open apex molars, systemic diseases, a history of root canal treatment, poor radiographic quality, and restorations, which were excluded due to their possible effect on pulp chamber morphology.

**Figure 1:**
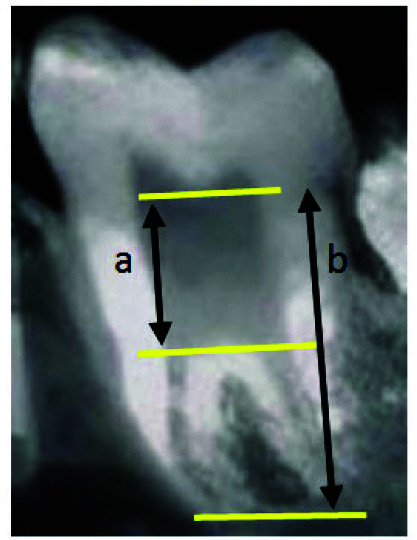
Taurodontic index used in the study: a/b 100×, **a:** The distance from the apical point in the pulp chamber roof to the coronal point in the pulp chamber floor, **b:** The distance between the pulp chamber roof and the longest root)

**Figure 2:**
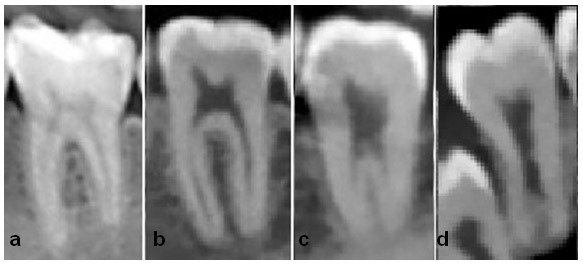
Radiographic views of taurodontic teeth **a:** Normal molar, **b:** Hypotaurodontism, **c:** Mesotaurodontism, **d:** Hypertaurodontism

**Figure 3:**
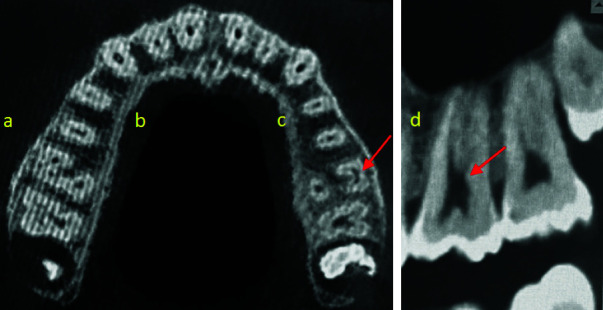
Representative images of C-shaped canal presence in a taurodontic maxillary first molar, the C-shaped canal was located in the buccal canals

Evaluating the CBCT images of patients and extraction of the required data from these images were performed anonymously by the project's executors using the NNT viewer software (NewTom, Venora, Italy). Initially, patients' demographic information (i.e., sex, and age) was recorded. Then, all radiographs were reviewed by executors until achieving agreements. The teeth were evaluated based on the criteria described by Shifman and Channel [ 5 ]. In the reconstructed panoramic, the distance from the apical point in the pulp chamber roof to the coronal point in the pulp chamber floor was measured and divided by the distance between the pulp chamber roof and the longest root, and the obtained value was multiplied by 100 (Figure 1). If the obtained value was equal or more than 20 and the distance from the CEJ to the pulp chamber floor was more than 2.5 millimeters, the respective tooth was classified as a taurodontic tooth. Then, the taurodontic teeth were classified based on their severity as follows: hypotaurodontism (mild form, with a value between 20%-29.9%), mesotaurodontism (moderate form, with a value between 30%-39.9%), and hypertaurodontism (severe form, with a value between 40% and 75%). (Figure 2) In addition, teeth were accessed for the C-shaped canal and its types in the axial plane. C-shaped canals were inspected according to the morphology at the levels of the pulp chamber floor and the extracted data and values were recorded in tables (Figure 3). 

### Statistical analysis

The statistical analysis was carried out using IBM SPSS software version 26.0. Qualitative variables were reported as frequency and percentage. In addition, the 95% confidence interval (CI) for prevalence rates was estimated using the bootstrap method. The correlation between qualitative variables and outcome variables was evaluated using the Pearson’s Chi-squared test or Fisher’s exact test. Moreover, the correlation between quantitative variables outcome variables was evaluated using the independent two-sample t-test or the Wilcoxon rank-sum test, depending on the distribution of the data. A level of less than 0.05 was considered statistically significant for the *p* Value. 

## Results

A total of 700 jaws (371 upper, 329 lower) were included in the study, with a median sample age of 40 years. Of the upper jaws, 193 (52%) belonged to male cases, while 177 (53.8%) of lower jaws belonged to males. Female patients accounted for 178 (48%) of upper jaws and 152 (46.2%) of lower jaws. The prevalence of taurodontism was significantly higher in the upper jaw, with 130 (35%) of upper jaws containing at least one taurodontic tooth compared to only 16 (4.86%) in lower jaws (*p*= 0.0001). A significant bilaterality was observed in taurodontism among the sample cases (84.7% vs. 15.3%) (*p*= 0.0001). The overall prevalence of taurodontism in teeth, regardless of jaw and tooth type, was calculated to be 9%, with 506 taurodontism found in 5600 inspected teeth. 

**Figure 4:**
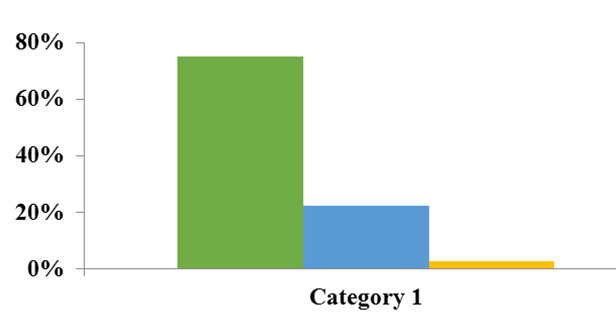
Prevalence of different types of taurodontism in sample cases (Green: hypotaurodontism, Blue: mesotaurodontism, Yellow: hypertaurodontism)

The prevalence of taurodontism according to tooth type is shown in Table 1. Figure 4 indicates that the majority of taurodontism cases are of the hypotaurodontic type, representing the mildest form of abnormality and comprising 75% of cases. 

**Table 1:** Taurodontism distribution according to the tooth type

Teeth	Right N(%)	Left N(%)	Total N(%)
Maxillary teeth			
First premolar	8 (1.1%)	8 (1.1%)	16 (2.2%)
Second premolar	24 (3.4%)	26 (3.7%)	50 (7.1%)
First molar	86 (12.28%)	88 (12.57%)	176 (25.14%)
Second molar	108 (15.42%)	104 (14.85%)	212 (30.2%)
Mandibular teeth			
First premolar	4 (0.57%)	4 (0.57%)	8 (1.1%)
Second premolar	6 (0.85%)	4 (0.57%)	10 (1.42%)
First molar	6 (0.85%)	4 (0.57%)	10 (1.42%)
Second molar	12 (1.7%)	12 (1.7%)	24 ( 3.4%)

There was no significant correlation between taurodontism and sex (*p*= 0.208), but individuals diagnosed with taurodontism were significantly younger ((36 years (IQR: 27, 44) vs. 41.5 years (IQR: 31, 53.75), *p*< 0.001)).

Among the 700 jaws, 106 (15%) contained teeth with C-shaped canals, distributed among 80 female and 26 male cases. A total of 391 teeth (6.9%) were detected with C-shaped canals, with the highest prevalence observed in second molar teeth (78 upper, 45 lower, totalinging 126) followed by first molar teeth (39 upper, 41 lower, totaling 77). Additionally, the occurrence of these canals was higher in the upper jaw (222 vs. 169) (Table 2).

**Table 2:** Distribution of C-shaped canals in teeth

Teeth		Maxilla (N%)	Mandible (N%)	Total (N%)
First premolar	Sample*	24 (3.2%)	43(6.5 %)	67(4.8%)
	Taurodontism*	11(68.8%)	5(62.5%)	16(66.7%)
	Normal*	21(2.9%)	32(5.0%)	52(3.8 %)
Second premolar	Sample*	81 (10.9%)	40(6.0 %)	121 (8.7 %)
	Taurodontism*	23 (29.2%)	5(50%)	28(46.7%)
	Normal*	22(3.17 %)	11(1.7%)	33(2.5%)
First molar	Sample*	39 (5.25%)	41(6.2%)	80(5.7%)
	Taurodontism*	31(17.6%)	5(50%)	36(19.4%)
	Normal*	8 (1.4%)	31(4.9 %)	39(3.2 %)
Second molar	Sample*	78 (11%)	45(6.8%)	123(8.8%)
	Taurodontism*	62 (29.2%)	15(62.5%)	77(32.6%)
	Normal*	16 (3.0%)	30(4.7%)	46(3.95%)

Sample*: distribution of C-shaped canals in sample teeth (overall), Taurodontism*: distribution of C-shaped canals in taurodontic teeth

Normal*: prevalence of C-shaped canals in normal teeth

C-shaped canals are significantly more detected in taurodontic teeth (*p*< 0.001). Type three was the most prevalent form of C-shaped canals in both normal and taurodontic teeth (46%), followed by type one (40%). 

## Discussion

This retrospective study was carried out on 700 Iranian gate the morphology of the pulp chamber and root canals of taurodontic teeth using CBCT images. We founda prevalence of 20.9% for taurodontism in the cohort, with a prevalence of 5.2% among the examined teeth. This rate was higher than that reported in most previous studies from Iran. It was reported to be 4.23% in the study of Farbiz *et al*. from Tehran [ 29 ], 5.7% in the study of Rakhshan *et al*. from Tehran [ 30 ], and 5.5% in the study of Bronoosh *et al*. from Shiraz [ 31 ]. However, the prevalence we found in Shiraz, located in southern Iran, was close to the 22.9% reported by Jamshidi *et al*. across eight cities in Iran [ 8 ]. Additionally, the prevalence of taurodontism was higher than in studies conducted in Trinidad and Tobago (11.28%) [ 19 ], India (ranging from 0.4% to 4.8%) [ 32 , 33 ], Turkey (0.26%) [ 34 ], Saudi Arabia (8%) [ 35 ] and Germany (2.25%) [ 7 ]. Nevertheless, the prevalence we found was lower than that reported in studies from China (ranging from 29.14 % to 52.4%) [ 36 - 37 ] and Brazil (up to 43%) [ 11 ] (Table 3).

**Table 3:** The prevalence of taurodontism and C-shaped Canal studied in the literature [7-8, 11, 19, 22, 29, 30-37]

Condition	Prevalence in Current Study	Comparison Studies
Taurodontism	20.9% (in cohort) 5.2% (in examined teeth)	Iran Studies: 4.23% (Tehran), 5.7% (Tehran), 5.5% (Shiraz)
		Trinidad & Tobago: 11.28%
		India: 0.4% to 4.8%
		Turkey: 0.26%
		Saudi Arabia: 8%
		Germany: 2.25%
		China: 29.14% to 52.4%
		Brazil: up to 43%
C-shaped Canals	15.4%	Middle Eastern (Lebanese): 19.1%
		South Asian (Burmese): 22.4%
		East Asian (Chinese): up to 41.27%
		East Asian (Korean): 31.3% to 45.5%

Such inter-study variation might arise from racial or genetic differences. Apart from racial disparities, factors such as sample selection, sample size, diagnostic criteria for taurodontism, imaging methods, and the inclusion/ exclusion of premolars and third molars influence the prevalence rate of taurodontism. Firstly, a smaller sample size leads to a less reliable prevalence rate. Secondly, many studies have used two-dimensional imaging techniques like intraoral periapical radiography and panoramic view to diagnose taurodontism, which may not accurately capture all parameters. Therefore, studies employing three-dimensional imaging techniques such as CBCT, like our study, may have higher detection ability in diagnosing taurodontism [ 38 ].Thirdly; an important consideration in epidemiological studies is that the prevalence of taurodontism tends to be lower when focusing on mandibular molars, as maxillary molars are more frequently affected. For instance, a Croatian study reported a 2% prevalence of taurodontism in mandibular molars, while a study on Brazilians including maxillary molars found prevalence rates ranging from 43% to 67% for different types of molars [ 10 - 11 ]. Fourthly, excluding third molars in some studies may result in underreporting of taurodontism, as these molars can also be affected by this anomaly. Some studies have indicated higher taurodontism rates in third molars [ 12 ]. Similarly, studies including premolars may show higher taurodontism prevalence [ 36 ]. Lastly, reporting prevalence based on the number of affected teeth rather than the number of patients can lead to a lower prevalence rate [ 31 , 34 ]. In this instance, the prevalence rate was 9% based on the total number of examined teeth, compared to 20.9% based on the number of patients.

In our study, taurodontism was significantly more prevalent in maxillary teeth than in mandibular teeth, with the highest prevalence in second (14.9% to 15.5%) and first (12.3% to 12.6%) maxillary molar teeth. This finding has been reported in several similar studies, including those by MacDonald Jankowski *et al*. [ 6 ], Tulensalo *et al*. [ 20 ] Gupta and Saxena [ 32 ], Patil *et al*. [ 33 ], Farbiz *et al*. [ 29 ], Jabali *et al*. [ 35 ], Li *et al*. [ 36 ], and Jamshidi *et al*. [ 8 ]. However, in several studies, particularly among Americans of European heritage and African Americans, mandibular second molars were the most affected by taurodontism [ 5 , 31 , 34 ]. Additionally, Mac-donald *et al*. [ 6 ] and Rao and Arathi [ 40 ] reported a higher prevalence of taurodontism in mandibular molars. This variation may be attributed to geographical factors.

Since taurodontism may be related to the X chromosome; its prevalence might be expected to be higher in women, as reported by some studies . However, similar to several other studies [ 1 , 5 , 7 , 8 , 12 ], we did not observe any significant difference between men and women regarding taurodontism prevalence, although it was slightly higher in women. Moreover, while some authors have suggested that such an insignificant sex difference might be due to the low prevalence of taurodontism in study populations with inadequate sample sizes, as well as a higher number of females in such studies, we did not find a significant difference despite a high prevalence of taurodontism, a relatively large sample size, and a higher proportion of females [ 8 ]. Therefore, it appears that other factors and genes may contribute to the occurrence of taurodontism. In addition, taurodontism type, laterality, and the involved tooth were not associated with sex. Similarly, Bronoosh *et al*. [ 31 ] found no significant differences regarding the type of taurodontism between males and females (*p*= 0.332). Additionally, Patil *et al*. [ 33 ] reported that they did not find a significant difference in the type of taurodontism between males and females (*p*> 0.05).

In our study, hypotaurodontism was observed as the most common morphological type. This finding was consistent with many studies [ 5 , 7 - 8 , 31 , 33 , 35 - 36 ]. Furthermore, we found that subjects with taurodontism were younger than those without taurodontism. Similarly, Jabali *et al*. [ 35 ] showed that the highest prevalence of taurodontism was in subjects aged 21-40 years, which was statistically significant, compared to the other age groups (*p*< 0.001). They proposed that such a finding could be attributed to the unequal sample.

With the use of CBCT, we were able to evaluate the morphology of complex root canals. We observed a prevalence of 15.4% for C-shaped canals. The observed rate was similar to a study from Middle Eastern countries (19.1% in Lebanese) and South Asian countries (22.4% in Burmese). However, it was lower than in East Asian populations (up to 41.27% in Chinese and 31.3%-45.5% in Koreans) [ 22 , 25 - 26 ] (Table 3). Additionally, the C-shaped canal was significantly more prevalent in those with taurodontism in our study. Although the association between taurodontism and CBCT is less studied, Aricioğlu *et al*. [ 27 ] found a high correlation between taurodontism and C-shaped canal configurations using CBCT. This association (coexistence of taurodontism and C-shaped canal configurations) can be important because in such cases, there is an endodontic challenge, and practitioners need to exercise caution during treatment due to the complexity of the canal system. The main limitation of the present study was its inclusion criteria of subjects who were referred to dental clinics. It is plausible that these subjects might have more dental anomalies, which could influence dental health. Therefore, our study might be subject to selection bias, lack generalizability, and introduce an overestimation of taurodontism prevalence. 

## Conclusion

This study revealed a noteworthy association between taurodontism and the presence of C-shaped canals, with individuals exhibiting taurodontic teeth significantly more likely to present this complex canal morphology (*p*< 0.001). While the overall prevalence of taurodontism in examined teeth was 9%, it was more common in maxillary molars and predominantly bilateral and mild in form. These findings underscore the clinical importance of recognizing taurodontism as a potential indicator of C-shaped canals, which can complicate root canal treatment. Enhanced awareness and diagnostic use of CBCT imaging can aid clinicians in early detection and more effective management of these challenging cases.
